# Patient and clinician perspectives of an eHealth intervention for supporting cancer treatment in the UK: mixed methods evaluation of the eRAPID randomised controlled trial

**DOI:** 10.1136/bmjopen-2023-078283

**Published:** 2024-11-07

**Authors:** Lorraine Warrington, Marie Holmes, Andrea Gibson, Rosemary Peacock, Zoe Rogers, Sarah Dickinson, Patricia Holch, Jenny Hewison, Claire Hulme, Bryony Dawkins, Barbara Woroncow, Virginia Cucchi, Eleanor Mae Hudson, Julia Brown, Galina Velikova, Kate Absolom

**Affiliations:** 1 Patient Centred Outcomes Research, Leeds Institute of Medical Research, University of Leeds, Leeds, UK; 2 Leeds Teaching Hospitals NHS Trust, Leeds, UK; 3 Leeds School of Humanities and Social Sciences, Leeds Beckett University, Leeds, UK; 4 Division of Health Services Research, Leeds Institute of Health Sciences, University of Leeds, Leeds, UK; 5 Department of Health and Community Sciences, University of Exeter Medical School, Exeter, UK; 6 Academic Unit of Health Economics, Leeds Institute of Health Sciences, University of Leeds, Leeds, UK; 7 Patient Representative, UK; 8 Leeds Institute of Clinical Trials Research, University of Leeds, Leeds, UK

**Keywords:** Oncology, Qualitative research, Telemedicine, Quality of Life, Surveys and Questionnaires, Patient Reported Outcome Measures

## Abstract

**Objectives:**

During 2015–2018, a randomised controlled trial (RCT) evaluated eRAPID, an eHealth intervention designed to capture patient-reported symptoms online during cancer treatment. eRAPID provides patients with advice on when to self-manage or seek medical support. Clinicians accessed symptom reports within electronic patient records. 508 participants starting systemic cancer treatment were recruited and followed for 18 weeks. The intervention group (n=256) was asked to access eRAPID and complete weekly online symptom reports. Clinicians received training on accessing and interpreting symptom reports. Overall, eRAPID had a positive impact on patients’ symptoms, quality of life and self-efficacy, particularly early in treatment and for patients with early-stage disease. Using mixed methods, we aimed to gather insight from patients and clinicians on how eRAPID worked to facilitate the interpretation of RCT findings.

**Methods:**

Following a concurrent triangulation design, patient experiences of eRAPID were gathered via end-of-study interviews (n=45) and questionnaires (n=186). Clinician experiences were obtained by end-of-study interviews (n=18) and completion, throughout the trial, of feedback questionnaires (n=787 from n=55 clinicians). Framework analysis was applied to examine qualitative data and close-ended questions were descriptively summarised. Findings were mapped against results from the RCT.

**Setting:**

Medical oncology services, UK cancer centre.

**Results:**

Patient feedback indicated eRAPID was easy to use. Adherence to weekly reporting was influenced by health status, reminders, perceived value and clinical use. Patient-reported benefits of eRAPID included an enhanced connection with the hospital, provision of practical advice and personal monitoring, which provided reassurance and empowerment. Clinicians were positive about the potential for online symptom monitoring but had mixed levels of direct experience with using eRAPID during the trial. Patients echoed this and recommended more explicit clinician use of symptom data.

**Conclusions:**

The mixed-method approach to capturing patient and clinician opinions provided valuable insight into the eRAPID intervention and complementary information on how the intervention was received and functioned.

**STRENGTHS AND LIMITATIONS OF THIS STUDY**

- The mixed-methods approach (combining results from interviews and feedback questionnaires) provides important insight into how the eRAPID health intervention functioned in practice when mapped to the findings from the main randomised controlled trial.
- The perspectives of a large number of participants involved in the trial were obtained (186 patients and 55 clinicians).
- Although feedback questionnaires were collected from clinicians throughout the study, interviews were only conducted at the end of the trial. The resources were not available for more objective assessments of how the intervention was used in practice (such as video or audio observations or system analytics).
- There are some biases in the study sample due to the trial eligibility criteria (English-speaking, basic level of computer literacy and internet access). In addition, it was difficult to capture the perspectives of those patients who did not engage as they often withdrew from the study.

## Introduction

 Systemic cancer treatments (chemotherapy, hormonal therapy, targeted drugs and immunotherapy) are associated with side effects affecting patients’ everyday functioning and quality of life (QoL) and can lead to life-threatening risks. Oncology teams are required to safely monitor patients during treatment to identify symptoms before they become serious, while providing advice for managing mild/moderate issues.^1 2^ As systemic treatments are typically administered in day-case outpatient settings, patients and caregivers play an important role in health monitoring from home but can have difficulty in determining the severity of issues.^3^ Standard practice for monitoring patients during treatments involves routine clinician-led assessment between cycles. Assessments rely on patient recall of issues experienced in previous weeks and clinicians making accurate judgements about severity. Standard practices do not easily allow comprehensive tracking of patient symptom trajectories over time.

There is a drive for health services to adopt technology-driven care solutions to improve cancer care during cancer treatment^4 5^ and growing international evidence demonstrates that electronic monitoring systems using patient-reported outcome measures (PROMs) in the cancer setting can benefit patient QoL^6^,^8^ and survival.^9 10^ However, electronic PROMs (ePROMS) to facilitate patient monitoring of symptoms have not been widely adopted^11 12^ and there is considerable variation in how systems are designed and embedded into clinical pathways.^13^ Patient and clinician views on everyday experiences of these systems are vital to understand mechanisms for intervention success and help refine development and implementation strategies.^14^

Developed using co-design principles, the eRAPID electronic health intervention allows patients to self-report symptoms online from home during treatment.^13 15 16^ eRAPID provides automated advice based on clinical algorithms to guide patients to self-manage mild/moderate issues or contact medical teams when potentially serious issues arise.

During 2015–2018, we evaluated eRAPID in a randomised controlled trial (RCT) in the systemic treatment setting with patients diagnosed with breast, gynaecological or colorectal cancer.^17 18^ The primary outcome was symptom control (measured by the Functional Assessment of Cancer Therapy Scale-General Physical Well-Being subscale^19^ (FACT-G PWB, scores 0–28, high scores=better symptoms) and secondary outcomes included PROMs to assess the impact on QoL and self-efficacy, in addition to the collection of the process of care data from hospital records (treatment delivery, hospital admissions and telephone contacts) and costs. Results evidenced better symptom control with eRAPID at 6 and 12 weeks, but not 18 weeks, from start of treatment. Improved patient self-efficacy to manage symptoms was found at 18 weeks. Benefits were more evident for patients with early-stage cancer than those with metastatic disease. Patient adherence to weekly symptom reporting was good with an average of 64.7% (varying between 72% in week 1 and 58% in week 18). eRAPID did not increase hospital workload or influence treatment delivery and the costs for the eRAPID group were lower at 18 weeks. Clinician use of symptom data was positively associated with patient adherence to online reporting, which was in turn associated with improved symptom control.^18 20^ However, use was variable between clinicians.

## Aims and objectives

As part of the RCT design, we adopted a mixed-methods approach to gain a better understanding of how eRAPID worked in practice.^21^ Interviews and questionnaires were used to elicit feedback and experiences from both patients and clinicians on their use of eRAPID and these results were combined and contrasted with the main RCT results.^22^ The aims were to:

- Explore patient and clinician views of the acceptability of eRAPID in terms of usability, value of specific system features and to identify how the intervention might be refined for future routine implementation.
- Explore barriers and motivators for the use of eRAPID for both patients and clinicians to inform future implementation.
- Better understand any benefits of eRAPID demonstrated in the RCT by exploring how the intervention impacted on clinical care.

## Methods

**Figure 1:**
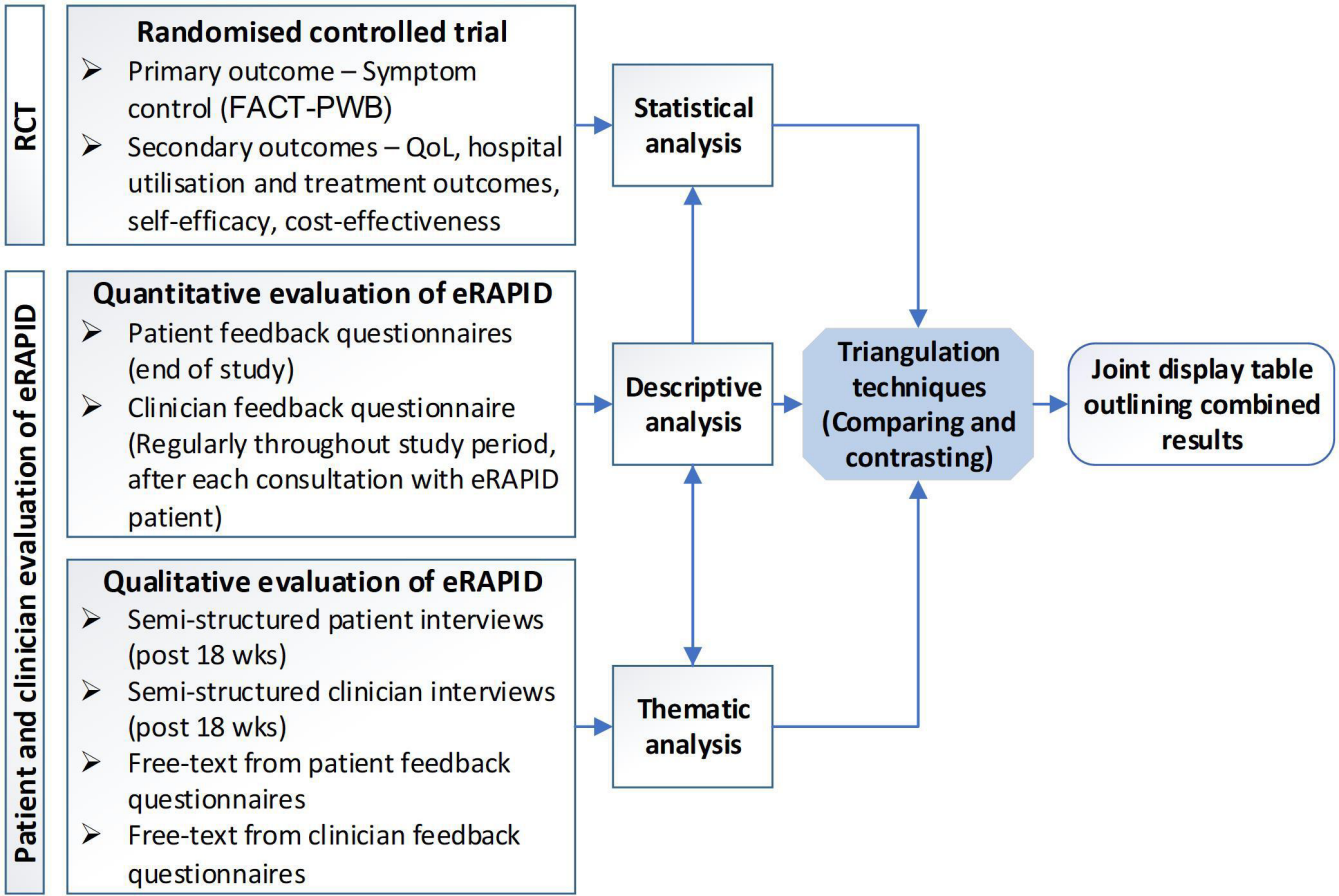
Overview of mixed-method approach using concurrent triangulation design. FACT-PWB, Functional Assessment of Cancer Therapy Scale-General Physical Well-Being subscale; QoL, quality of life; RCT, randomised controlled trial; wks, weeks.

We used a concurrent triangulation design,^23^ combining both qualitative and quantitative data from patients and clinicians evaluating eRAPID, with the results of the RCT (figure 1). More detail on the data and analysis techniques used is outlined below.

### eRAPID RCT in systemic cancer treatment

Full details of the eRAPID intervention and RCT are described in the published protocol.^17^ In summary, this was a single-site parallel RCT with an internal pilot in a UK cancer centre. English-speaking adult patients with internet access starting systemic treatment for breast, gynaecological or colorectal cancer were eligible. Participants were randomised to usual care or eRAPID intervention plus usual care.

Intervention participants had access to eRAPID and were asked to complete symptom reports online (via computer, tablet or smartphone) weekly for 18 weeks (reminders sent via SMS or email). The system provided automated severity-tailored patient advice for managing reported issues. Mild or moderate issues generated self-management advice and/or recommendations to discuss the issue at the next clinical visits. For severe and clinically relevant symptoms, patients were advised to immediately contact the 24-hour acute oncology service. Email notifications were sent to key clinicians; however, this functionality was not highlighted to patients, to avoid creating an expectation of direct follow-up. Patients could view graphical summaries of their symptoms over time. Clinicians were trained to access and interpret patients’ symptom reports which could be accessed within the hospital’s electronic patient records (EPR) and viewed in tabular or graphical formats.

### Procedures for obtaining feedback from patients and clinicians

#### Interview procedures

Patients: We invited a subsample of intervention participants to complete an interview at the end of the study period (18 weeks). We aimed to interview 5–10 per cancer site and purposively sampled participants based on age, sex, cancer site and adherence to weekly symptom reporting. Patients were interviewed at their convenience at the end of the study, in a private room at the hospital. The semi-structured interview schedule was originally developed based on concepts influencing behaviour change, such as motivators, barriers, attitudes and intentions. This was piloted in a usability study^24^ and some minor refinements were made. Broadly, the interviews explored personal experiences, use and views of eRAPID, impact on medical care and interactions with clinicians (online supplemental file A).

Clinicians: We arranged end-of-study interviews with up to five clinicians (specialist nurses and oncologists) from each cancer site. The semi-structured interview schedule (online supplemental file B) explored access and use of eRAPID patient data and its perceived value in clinical practice.

### Feedback questionnaire procedures

We obtained additional feedback through questionnaires.

#### Patient feedback questionnaire

We developed a feedback questionnaire to complement the data captured in the interviews. All patients on the intervention arm who were still on the study at the end of 18 week period were invited to complete this, allowing us to gain feedback from a wider range of patients. The questionnaire included:

- 12 closed questions focusing on the ease of using eRAPID, how symptom data were used by the clinical team, and the perceived value of eRAPID for themselves and future patients (online supplemental file C).
- Five free-text questions covering the use of eRAPID:
  - Reasons for non-adherence to weekly reporting.
  - Positives and negatives.
  - Suggestions for improvement.
  - Any other comments.
- The System Usability Scale (SUS).^25^ A 10-item scale is widely used to gain a subjective assessment of the usability of computer systems. Participants rate 10 statements from 1 to 5 (strongly agree to strongly disagree). Overall scores range from 0 to 100 with higher scores indicating better usability. Scores over 68 are above average.

#### Clinician feedback questionnaire

Clinicians were prompted to complete feedback questionnaires throughout the 18 week study period, each time they had a routine consultation with an eRAPID intervention patient. This questionnaire was developed by the research team for use in a previous RCT assessing clinician use of PROMs in clinical practice^26^ (online supplemental file D).

The questionnaire included:

- Close-ended questions to indicate if and how clinicians:
  - Used eRAPID data.
  - Found eRAPID useful.
  - Used eRAPID to contribute to patient management.
- Free-text boxes to provide comment on:
  - Additional ways they found eRAPID useful.
  - Any other comments.

### Patient and public involvement

Patient and public involvement (PPI) was prioritised throughout the eRAPID programme of work and further details of this are available in the published report.^20^ In the work described here specifically, our PPI coauthors (BW and VC) have supported the development of evaluation methods, reviewing patient materials such as information sheets and questionnaires and contributed to manuscript preparation.

### Analysis

#### Qualitative data (interviews and free text written comments)

Interview recordings were transcribed verbatim, transferred to NVivo and analysed using a framework method by members of the eRAPID research team (KA, LW, MH, RP, AG, ZR, SD). The framework method is a type of thematic analysis that can be applied using a combined deductive and inductive approach. This approach allowed the team to answer the specific research questions while allowing for the discovery of unexpected themes and topics.^27 28^ Following data familiarisation, we created a coding framework guided by the topics in the interview schedule and subthemes identified from the data. Two researchers coded each transcript and the team worked collaboratively to resolve queries, refine the framework and maintain a coding log. We allocated one or more main themes to each researcher to extract relevant coded quotes from NVivo into separate spreadsheets for charting and summarising data to draw overall conclusions. We collated, reviewed and summarised free-text responses from feedback questionnaires under the overarching qualitative coding framework.

#### Quantitative data (close-ended questions)

We conducted an analysis using SPSS V.26. We scored the SUS according to instructions. Differences between cancer sites and metastatic and non-metastatic patients were explored using one-way analysis of variance (ANOVA) and independent t-test, respectively. Close-ended responses from feedback questionnaires were summarised using descriptive statistics.

#### Synthesis of participant feedback with main RCT findings

Using the joint display approach to integrating qualitative and quantitative data in mixed methods studies, we mapped patient and clinician feedback against the primary and secondary eRAPID RCT outcomes.^22 29^

## Results

### Participants

#### Patient sample

Target recruitment was met with 508 patients consented and randomised in the RCT: usual care (n=252) and eRAPID intervention (n=256). 222 patients in the intervention arm remained in the study for 18 weeks and 186 (84%) completed feedback questionnaires and 45 participated in interviews (table 1). 20% (n=38/186) of the patients who completed feedback questionnaires and 24% (n=11/45) of the patients interviewed had previously had chemotherapy.

**Table 1:** Overview of participants who completed interviews and feedback questionnaires

Patients		Interviews* (n=45)	Feedback questionnaires* (n=186)
Age	Mean age, years (SD)	54.6 (12.5) range 22–80	57.0 (11.7) range 24–86
Sex	Male	9 (20%)	43 (23%)
	Female	36 (80%)	143 (77%)
Breast	Total	24 (53%)	87 (47%)
	Primary/local	23 (96%)	83 (95%)
	Metastatic	1 (4%)	4 (5%)
Gynaecological	Total	9 (20%)	34 (18%)
	Primary/local	2 (22%)	6 (18%)
	Metastatic	7 (78%)	28 (82%)
Colorectal	Total	12 (27%)	65 (35%)
	Primary/local	9 (75%)	35 (54%)
	Metastatic	3 (25%)	30 (46%)
**Staff**		**Interviews (n=18**)	**Feedback questionnaires (n=55**)
Category	Specialist nurse	7 (39%)	10 (18%)
	Senior oncologist	8 (44%)	15 (27%)
	Junior oncologist	3 (17%)	28 (51%)
	Pharmacist	0	2 (4%)
Clinic	Breast	6 (33%)	19 (35%)
	Gynaecological	6 (33%)	14 (26%)
	Colorectal	2 (11%)	8 (15%)
	Mixed clinics	4 (22%)	14 (26%)
Sex	Female	12 (67%)	38 (69%)
	Male	6 (33%)	17 (31%)

* These are not distinct groups. Some participants who completed interviews also completed feedback questionnaires.

#### Clinician sample

55 clinicians participated in the RCT, using eRAPID data during routine consultations, all completed at least one feedback questionnaire and 18 were interviewed (table 1). Of an expected 1314 questionnaires, 787 (59%) were completed and 218/256 (85%) of intervention patients had their symptom data reviewed by a clinician at least once.

Reasons for questionnaire non-completion included clinicians forgetting due to the relatively small number of eRAPID intervention patients seen in clinics, researchers being unable to prompt clinicians due to last-minute appointment changes and clinicians not having symptom data to review due to patient non-adherence.

### Patient perspectives

Patient interviews and feedback questionnaires covered three overarching and interlinking themes:

- Acceptability and functionality
- Impact on clinical care.
- Personal value of using eRAPID.

**Figure 2:**
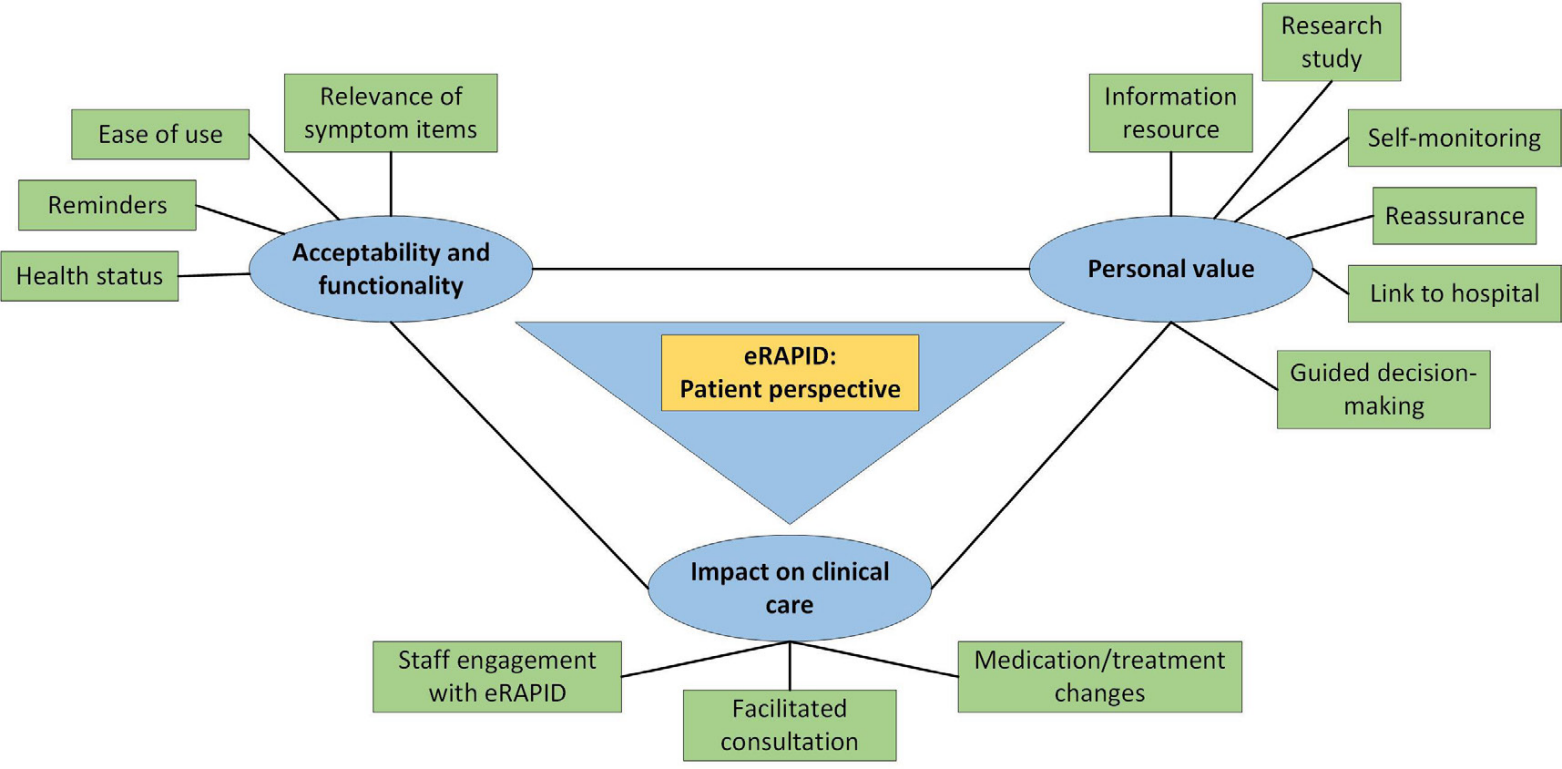
Overview of patient perspective of the use and impact of eRAPID.

We describe each theme below with a focus on patients’ views on the use of eRAPID. Figure 2 provides a graphical representation summarising key elements of the patient perspective.

### Acceptability and Functionality

This theme explored how easy patients found the navigation and use of eRAPID to complete their symptom reports and what the main barriers and facilitators were for adherence to weekly symptom reporting.

#### Ease of use

**Figure 3:**
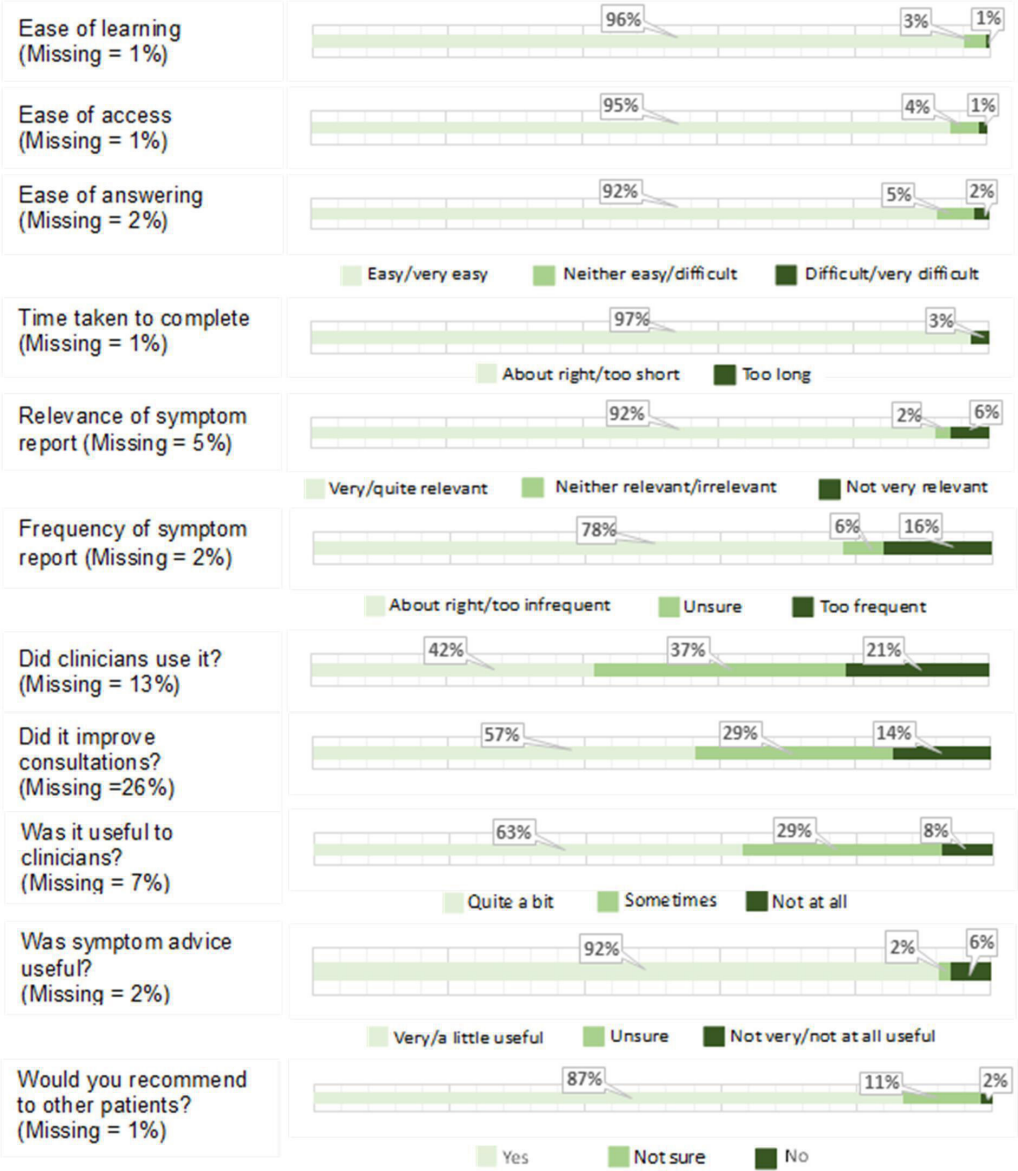
Feedback on eRAPID from patient questionnaires. Gynae, gynaecological.

Quantitative data from feedback questionnaires (figure 3) indicated most patients found eRAPID easy to use (96%), easy to complete (92%) and thought the length of time it took was about right (97%).

SUS scores ranged from 25 to 100 with a mean of 83.3 (SD 14.4). An independent t-test indicated patients with non-metastatic disease reported higher scores (M=86.0, SD=12.8) than those with metastatic disease (M=80.7, SD=16.9) and this was statistically significant (95% CI, p=0.036). A one-way ANOVA (*F* (2,173) = 2.919, p=0.057) indicated no statistically significant difference in SUS scores between patients with breast (M=87.0, SD=12.8), colorectal (M=81.4, SD=16.9) and gynaecological (M=83.0, SD=11.7) cancer.

Interview data also indicated that patients found eRAPID easy to use and did not experience any major issues accessing or using the system. Comments from free-text sections of the feedback questionnaire suggested some improvements including the creation of an eRAPID application and the provision of the facility to provide more detailed information about symptoms, upload photos for specific symptoms such as rashes, and record current medications.

#### Reminders

Email/text reminders were important facilitators for adherence, though some individuals also set their own weekly routines.

‘… I’d kind of disciplined myself to do it on a Wednesday.’ (Patient A, Gynaecological)

#### Health status

Health issues such as fatigue, cognitive/memory issues and hospitalisation were common barriers to adherence.

‘…it was nothing to do with the system or finding it difficult, the thing that was difficult for me was the absolute fatigue with the chemotherapy, just totally wiped me out.’ (Patient B, Gynaecological)

#### Relevance of symptom items

Patients found the symptom report relevant (92%) and qualitative data supported this. However, some found the weekly completions and associated advice repetitive, particularly when their symptoms did not change. Some thought the response options were too limited and did not allow scope to add detail.

‘The answers could be too black or white, when life is generally more grey and there were no extra boxes to explain.’ (Patient C, Colorectal)

### Impact on clinical care

This theme explored patients’ perceptions of how eRAPID impacted on their clinical care and influenced their interactions with clinical staff during their cancer treatment.

#### Clinician engagement with eRAPID

42% of patients thought clinicians regularly used their symptom reports while 21% thought they were not used at all. Qualitative comments supported these mixed experiences. A few patients reported clinicians being explicit about using eRAPID data to guide consultations.

‘…our chemotherapy doctor, he would bring it up every time and show us it and talk me through any concerns that he had… that re-incentivised me to use the system because you know it’s not just a waste of time, somebody’s looking at it.’ (Patient D, Gynaecological)

However, others expressed significant disappointment that clinicians did not use their symptom reports and cited this as a barrier to use. A clear recommendation from patients for future refinement of eRAPID was increased and explicit clinician use of the symptom reports.

‘No feedback from anyone—was expecting at least someone discussing usage of system but didn’t happen at all after using it for 3 times—so stopped using it.’ (Patient E, Colorectal).

#### Facilitated consultations

63% of patients thought their symptom reports were useful for clinical staff, often leading to a better understanding of experiences. Weekly symptom reporting served as a memory prompt, as patients did not have to try to recall symptoms weeks later.

‘At clinic visits I had sometimes forgotten about some of the symptoms I had experienced over the three week period since my last visit…’ (Patient F, Breast)

#### Medication/treatment changes

Some patients described changes to their clinical management, such as prescription of medications or changes to their chemotherapy, as a direct result of their symptom reports.

‘Doctors and nurses referred to my answers. Doctor reduced chemo dosage to help my sore throat.’ (Patient G, Breast)

### Personal value of using eRAPID

This theme describes the range of personal benefit patients experienced from using eRAPID.

#### Link to the hospital

Some patients experienced a heightened sense of connection with the hospital:

‘It helps with continuity of care. I feel under constant supervision of my treatment.’ (Patient H, Breast)

‘It’s like keeping in touch… without making an appointment to see anyone.’ (Patient I, Colorectal)

#### Information resource

Patients found the symptom advice useful (92%). Many reported reassurance in having tailored advice from a trusted source and having their symptoms monitored.

‘Peace of mind that you were being monitored and any potential issues for example, high temperature would give you guide as to whether to ask for help.’ (Patient J, Breast).

For some metastatic patients who had chemotherapy previously, the value of advice was limited as they were already familiar with how to manage symptoms.

‘Well because I’m a bit of an old hand at chemo I think….it was only telling me what I already knew.’ (Patient K, Gynaecological)

#### Self-monitoring

The process of routine symptom reporting and tracking symptoms over time was also empowering.

‘Felt good to record my symptoms every week—felt like I was taking an active role in my treatment.’ (Patient L, Breast).

‘I think it was useful for us because you got the little graphs. So, you could compare how you… were feeling in comparison to how you’d been before.’ (Patient M, Colorectal)

For some the benefit of the system was more apparent early on in treatment and less useful later as they became familiar with symptoms/treatment.

#### Guided decision-making

In some cases, the symptom advice engendered a sense of confidence that patients and carers were taking the right action, including when to seek medical advice:

‘…gave me and my family more confidence to manage side effects especially early on in the treatment… gave me ‘permission’ to contact the hospital if I was worried….’ (Patient O, Colorectal)

#### Research study

Some patients reported that their main motivation for adherence was a sense of responsibility to honour their commitment to participating in the research, rather than personal benefit.

‘I saw it as, ‘well I have agreed to this research thing so I will do it’…So that’s probably the biggest motivator… just because I said I would do it.’ (Patient P, Gynaecological).

### Clinician perspectives

Clinician feedback on eRAPID was summarised into the following overarching themes.

- Acceptability and functionality.
- Impact on clinical care.
- Perceived value of eRAPID for patients.

The main descriptive results from clinician feedback questionnaires are included in the themes below. Additional findings are in online supplemental file E.

### Acceptability and functionality

This theme explored clinicians’ views on how easy it was for them to view, access and interpret patients eRAPID reports. Predominantly clinicians found it easy to access symptom reports within the EPRs.

‘The system was very easy to use, it’s on the system we use in clinic, you just have to click a button, all the information is there, so it was easy to use, readily available.’ (Colorectal, Senior oncologist)

The presentation of symptom data in both tabulated and graphical forms was useful to address different needs and preferences.

‘I quite liked the graphs, simply because it was very quick and easy to be able to see if something had particularly changed.’ (Gynaecology, Senior oncologist)

‘I like the tables, I’m not a big fan of the graphs… it’s easier to see quite a lot of information quickly on the tables…. Personally, I didn’t see the extra value to the graphs.’ (Colorectal, Specialist nurse)

Due to the relatively small number of eRAPID intervention patients seen in clinics, it was easy for clinicians to miss reports, particularly as there was no facility in the electronic records to flag them.

‘I think it will be even more useful when, if it’s used in routine practice because you wouldn’t forget to look at it.’ (Colorectal, Senior oncologist)

#### Impact on clinical care

This theme describes clinician views on if and how eRAPID impacted on patients’ clinical care and influenced their decision-making.

**Figure 4:**
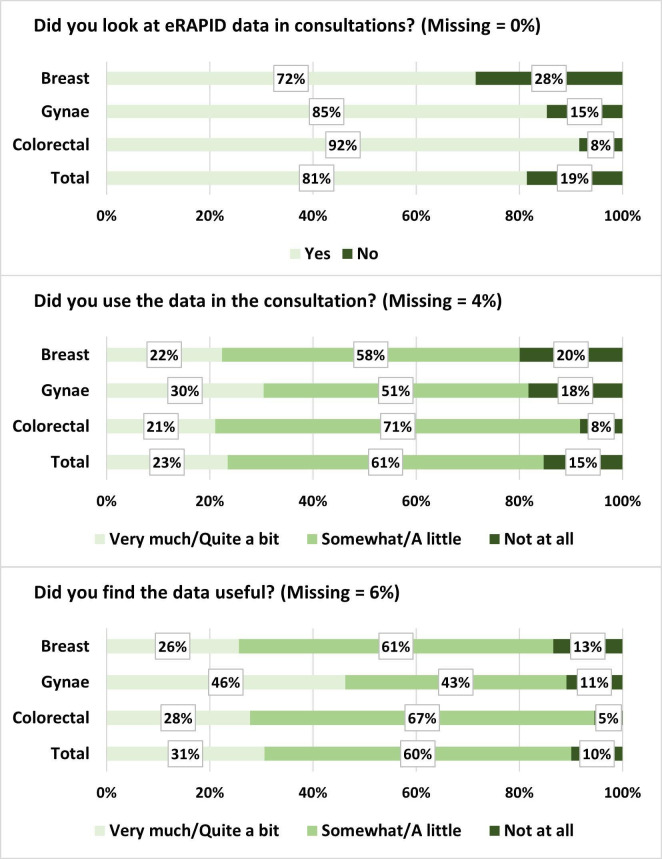
Feedback on eRAPID from clinician questionnaires.

Clinicians reported accessing eRAPID data on 81% (641/787) of the post-consultation feedback questionnaires completed. Clinicians rated to what extent they used eRAPID and how useful they found it on a Likert-type scale from ‘not at all’ to ‘very much.’ 90% used it at least ‘a little’ and 90% found it at least ‘a little’ useful (figure 4).

Gynaecology clinicians were more likely than breast or colorectal clinicians to report using eRAPID ‘quite a bit’/‘very much’ (30% vs 22–21%) and finding data useful ‘quite a bit’/‘very much’ (46% vs 26–28%). However, gynaecology and breast clinicians were also more likely to report not using the data at all (20–18% vs 8%) and not finding the data at all useful (13–11% vs 5%) compared with colorectal clinicians.

Clinicians indicated finding eRAPID useful on 663/787 (84%) of feedback questionnaires. Those that answered ‘Yes; to this question were asked to indicate the specific way or ways it was used from a list of options, 51% said it confirmed knowledge of patients’ issues, 26% said it provided additional information, 23% said it identified issues to discuss and 8% said it contributed to management (online supplemental file E).

Qualitative interview data supported these findings with clinicians describing eRAPID as a helpful tool in structuring/preparing the consultation and building a connection with the patient.

‘I found it helpful because it informs you before the patient arrives and I think it also stops you having to ask the patient 300 questions every time they come.’ (Gynaecological, Specialist nurse)

‘There is an instant rapport because she thinks okay this one knows about me and I think that’s been very helpful for me.’ (Breast, Senior oncologist)

However, other clinicians thought using symptom reports made consultations longer. One clinician found using eRAPID to be a conflict to their usual practice.

‘… you have your own way of doing it, which I’ve been doing for such a long time and I just, it just didn’t kind of resonate with me I’m afraid.’ (Breast, Senior oncologist)

Clinicians recognised the benefit of being able to identify trends in symptom trajectories and viewed the symptom reports as accurate. However, some had reservations about patients reporting issues not relevant to the cancer/treatment and some reported a lack of concordance between what patients reported online versus face-to-face.

‘Patient contradicted information reported on eRAPID, that is, denying any nausea which was confusing.’ (Colorectal, Specialist nurse)

In a relatively small number of consultations (n=56), clinicians indicated that eRAPID contributed to management, such as a change to chemotherapy/medication (online supplemental file E). Qualitative data supported this, as some clinicians reported using eRAPID data to make decisions such as prescribing antibiotics for infections, providing advice on laxatives and reducing chemotherapy doses.

‘Enabled to advise regular antiemetic and anti-spasmodics based on their pattern of occurrence relating to chemotherapy cycle.’ (Breast, Specialist registrar)

### Perceived value of eRAPID for patients

This theme explored clinician views of if and how eRAPID was useful for patients during cancer treatment. Several clinicians commented that eRAPID was beneficial for patients.

‘…it gave them permission to ring when they potentially may have not necessarily rung but may have tolerated it to the point where it becomes just slightly less easy to resolve.’ (Breast, Specialist nurse)

However, others described a range of patient-centred barriers to adopting the system into routine care, which included variation in patient compliance with online reporting, requirement of English language and IT access and fluency.

‘……the patients that don’t have access to the computer are the patients that we should be more concerned about because they might be…less literate or …less able to communicate their needs and concerns*…*’ (Colorectal, Specialist nurse)

### Synthesis of feedback with key findings from the eRAPID RCT

In table 2, we present the key RCT findings and map these with experiences described by patients and clinicians during interviews and in feedback questionnaires.

**Table 2:** Synthesis of feedback with key findings from the eRAPID RCT

Key findings from RCT^18^	Relevant themes from qualitative data	Summary of patient and clinician experiences	Level of complementary evidence
eRAPID associated with better: Symptom control (FACT-G PWB) at 6 and 12 weeks. Health status and overall QoL at 18** **weeks.	Personal value of using eRAPID (subthemes: link to the hospital, Information resource, self-monitoring, guided decision-making, research study).Acceptability and functionality (subthemes: ease of use, reminders, health status, and relevance of symptom items)	Patients reported examples of where the intervention: Supported personal decision making to seek medical advice/manage symptoms. Provided reassurance and valuable information. Was more useful in the early weeks of chemotherapy.	Good supporting evidence
eRAPID associated with better self-efficacy for symptom management at 18 weeks.		Patients found aspects of the intervention ‘empowering’ and felt like it gave them an active role in their care.	Good supporting evidence
Positive benefit of eRAPID observed in non-metastatic cancer group only.		Metastatic group reported lower system usability scores.Some metastatic patients found the symptom information and advice less useful to them as they had been through chemotherapy before.	Some supporting evidence
Patient adherence to symptom reporting was positively associated with clinicians' reported use of eRAPID reports.No differences observed between arms for chemotherapy delivery, hospital admissions, acute oncology assessments or emergency hotline calls.	Impact on clinical care (subthemes: clinician engagement with eRAPID, facilitated consultations, medication treatment/change)	Patients had mixed experience of staff use of their symptom reports.Some patients reported that eRAPID gave them ‘permission’ to call the hospital with symptoms. However, patients also reported not completing symptom reports when they were very unwell.Some clinicians described using the eRAPID data to make decisions on chemotherapy and/or supportive medications. However, clinicians varied in how often they reported using the data and how useful they found it.	Some supporting evidence
Adherence to weekly eRAPID online reporting was good.Adherence reduced over time with patients completing less consistently towards the end of the 18 week period.Some participants completed none or very few reports.	Acceptability and functionality (subthemes: ease of use, reminders, health status and relevance of symptom items)	Patients reported that the online reporting was easy to use.Scores from the System Usability Scale were high.Patients also reported that eRAPID was most useful in the initial weeks of treatment.Reasons given for non-adherence to completing symptom reports were forgetting, ill health and not finding the reports as useful/too repetitive over time.	Good supporting evidence

FACT-G PWBFunctional Assessment of Cancer Therapy Scale-General Physical Well-Being subscaleRCTrandomised controlled trial

### Improved symptom control (FACT-G PWB) at 6 and 12 weeks, health status and overall QoL at 18 weeks and self-efficacy at 18 weeks

Patient feedback supported our findings of the benefits of eRAPID with patients reporting detailed examples of how the intervention was beneficial. Qualitative findings offered insight into why the benefits of the intervention were limited to the earlier stages of treatment, for example, lack of impact on symptom control at 18 weeks. Patients often reported finding symptom advice more useful during the initial weeks of chemotherapy and less useful later as they became more experienced in symptom management. Some metastatic patients with previous chemotherapy experience reported that eRAPID would have been more useful the first time around, offering insight into the greater benefits seen in the non-metastatic patient group.

### High rates of patient adherence

Qualitative data indicated that eRAPID was easy to use and access. However, in some instances, adherence declined towards the end of the 18 weeks. Again, this may be explained by some patients finding eRAPID less useful in later stages of chemotherapy. Additionally, patient adherence was associated with the reported clinician use of eRAPID during consultations. Qualitative feedback from patients reported explicit clinician use of eRAPID as a motivator for engagement, but a barrier when clinicians did not acknowledge their symptom reports.

### No impact of eRAPID on chemotherapy delivery, hospital admissions, acute oncology assessments or emergency hotline calls

Clinician feedback questionnaires reported a small number of examples of using eRAPID data to guide treatment decisions, however not enough to expect to see an impact on treatment delivery. Patients reported that eRAPID gave them ‘permission’ to contact the hospital for severe symptoms; however, they also reported that self-management advice empowered them to manage symptoms at home, indicating the complexity of the impact of eRAPID on hospital utilisation.

### No difference in benefits of eRAPID between breast, colorectal and gynaecological patient groups

Qualitative data indicated some differences in how eRAPID was used in the different groups. For example, there were differences in clinician engagement, with gynaecological clinicians typically engaging more with eRAPID. However, the metastatic patients who had higher representation in the gynaecological group, also reported finding the self-management advice less useful due to having previous experience with chemotherapy.

## Discussion

As part of the eRAPID RCT, we aimed to capture information from patients and clinicians, via interviews and written feedback, to understand experiences of using the system to help explain results and improve future refinement of this approach in cancer care.

Both patients and clinicians reported that eRAPID was easy to use. The main advantages from a patient perspective included its role as a trusted source of information and advice, providing an enhanced connection with the hospital. However, patients thought the system could be improved, particularly in terms of clinician use. Although some patients reported that clinicians actively addressed and used their symptom reports, others had no recollection of clinicians reviewing their data at all. Understandably, this was disappointing leading to some patients becoming less engaged. These findings align with results from the RCT where clinician use of data was positively associated with patient adherence to weekly completions.

In addition, we found important benefits for patients around increased self-efficacy and QoL in the RCT. Previous trials have focused on patients with advanced disease and our findings demonstrating the benefits of this approach for patients with early disease is an important one. The qualitative insight we have gained about the mechanisms of this benefit has valuable implications for the future development and implementation of similar systems.^14^

Some clinicians were very positive about the value of eRAPID for assisting with consultation preparation and providing a focused discussion. Some found it valuable in saving time and identifying symptom trends. In practice, the design of the RCT meant some clinicians had limited exposure to eRAPID intervention patients, and the lack of an automated facility for flagging reports in the EPRs meant they could easily miss patients with symptom reports available.

Clinician feedback was variable between clinics, with those in gynaecology reporting higher use and usefulness of eRAPID. However, this did not translate into a difference in outcomes between patients in the different cancer sites. This may be simply because our RCT was not powered to detect statistical differences in secondary outcomes such as these, and it may also be partially due to differences in how individual clinicians used data or the complex multifaceted ways eRAPID benefitted patients. For example, the RCT indicated the intervention was more beneficial for non-metastatic patients and qualitative data provided some insight into this, with patients who had experienced chemotherapy previously finding the information less novel/useful. The gynaecological group had a high proportion of metastatic patients, particularly in comparison to the breast group. While gynaecology patients may have benefitted from increased clinician engagement, this advantage may have been diminished by the higher proportion of metastatic patients when compared with the colorectal and breast clinics who seemed to derive greater benefit from the eRAPID information and advice.

Evidence from other trials has indicated that remote monitoring can impact outcomes such as hospital admissions, treatment delivery and even survival.^6 10^ This was not a finding in our RCT, which did not find a difference between eRAPID and usual care for hospital contacts or admissions. Although our qualitative data indicated that eRAPID guided patient decision-making about hospital contact and self-management, it is likely that the impact of eRAPID on hospital contacts is complex, and difficult to assess by a quantitative comparison. eRAPID may increase the number of contacts and admissions by advising patients to contact the hospital, while on the other hand, it may reduce contacts by supporting self-management when appropriate.

There are some limitations to our methods and the scope of findings. First, we conducted patient interviews at the end of the study period. Longitudinal interviews over the course of the 18 week study period may have provided more understanding of how patient use and engagement with eRAPID fluctuated over time. However, the interview data did provide some nuanced insights into patient and clinician experiences of how eRAPID impacted care. Second, we relied on patients and clinician accounts of how eRAPID symptom reports influenced care. Clinicians usually completed feedback questionnaires immediately after consultations; however, we only collected basic information due to clinical time constraints. In addition, there was a high rate of missing data for these questionnaires, limiting their generalisability. In a previous study, we found it useful to audio-record consultations and use coding methods to evaluate how PROMs influenced discussions.^26^ However, this was not possible in the current study due to resource constraints and the pragmatic nature of the trial.

Another limitation is that patients who did not engage with eRAPID at all were likely to withdraw from the trial and were unavailable for interview or questionnaire completion. However, this was a relatively low proportion of patients and we specifically targeted those with low adherence to compensate for some of this bias. There will be some additional bias in our sample simply because eligibility required patients to be English-speaking and to have some level of information technology (IT) skills and access.

Moving forward we are working on future implementation strategies to take eRAPID into routine care. We have experienced similar challenges around implementation to those reported by others working in this arena across clinical areas,^12^ such as barriers around hospital IT systems and healthcare infrastructure. An important element of ongoing work is the engagement and training of both patients and clinicians to maximise the use and clinical value of PROMs data. Ensuring that selected PROMs are both relevant to clinical care and meaningful to patients while managing the burden of item completion remains challenging. Ongoing efforts to explore how PROMs content should be refined to align with clinical need through the cancer trajectory and the potential for incorporating computer adaptive testing techniques are warranted. Insights provided by this qualitative work and our previous development activities is vital to contribute to an evidence base of patient and clinician perspectives in a variety of contexts and give insight into how to successfully implement ePROMs into the clinical pathway.^30^,^32^ We have funding to expand on the analysis of the eRAPID study data using innovative methodologies such as through case study and latent class analysis, in addition to exploring optimal methods of PROMs data visualisation for both clinicians and patients. This work will further inform the clinical value of PROMs data in cancer practice and enable targeted refinement of the eRAPID intervention.

As PROMs become more widely adopted, it remains vital to explore their practical implementation to ensure they effectively serve patients and clinicians. ePROM interventions like eRAPID, are often complex and multifaceted. Qualitative methods used alongside evaluations can provide invaluable insight into the mechanisms by which patients and clinicians may benefit and identify limitations and opportunities for improvement.

## supplementary material

10.1136/bmjopen-2023-078283 online supplemental file 2

## Data Availability

Data are available upon reasonable request.
