# Supplementary material for: Patient and clinician perspectives of an eHealth intervention for supporting cancer treatment in the UK: mixed methods evaluation of the eRAPID randomised controlled trial
Source: BMJ Open. 2024 Nov 7;14(11):e078283. doi: 10.1136/bmjopen-2023-078283 (PMC11552561; doi:10.1136/bmjopen-2023-078283)

Appendices and Supplementary files

Supplementary file A: Summary of patient Interview schedule

General views on using the system e.g.

- Did you have any problems accessing eRAPID at any time? Did you find it easy to use?
- Has it been difficult for you to complete the questionnaire on a weekly basis? Is there anything we could do to make this easier for you or other patients?
- What do you think the main value of eRAPID would be for patients? Were there any advantages and/or disadvantages to using eRAPID?
- Would you be happy to use eRAPID again in future if you had the need to?

Completion of symptom reports

If patient initially started using the system but then stopped.

- You initially used the system regularly but then you stopped. Can you remember the reasons why this was?
- Did you intend on using the system again in the future?
- Is there any support we could have given you to help you to complete at this time?

If patient has completed intermittently

- You used the system intermittently throughout the study. Can you remember the reasons why you didn’t complete at this time?
- Is there any support we could have given you to help you to complete at this time?
- What made you start using the system again?

If the patient used the system regularly throughout the study.

- You used the system regularly. Can you tell us what your main motivations were for doing this? (For example, the graphs, self-management advice or for the clinicians) Did you feel that it helped you? If so, in what way?

Self-management advice

- Do you think that the system accurately assessed your symptoms? E.g. the types of questions asked, the severity level, etc.
- Did you get advice on how to manage your symptoms? Was it helpful? In what way?
- Did you receive advice to contact the hospital at any point? Did you think it was appropriate? Did you follow this advice? If not, what were your reasons for not following the advice?
- Did you find the information on the eRAPID website useful? Did you use any of it? Do you think that using the system had any effect on how you managed your symptoms and side-effects?

Graphical summaries of symptom reports

- Did you look at/use the graphs at the end of questionnaire?
- If not, can you tell us the reason (e.g. didn’t find them useful, too complicated)
- If so, did you find them useful? In what way? What did you like about them? What did you not like about them?

Staff use of symptom reports

- Did the doctors/nurses use the system at your clinic appointments? What do you think the main value would be for clinicians?
- Do you think that using the system influenced your consultations in any way? If so, how? E.g. Do you think you had any medications prescribed or changes in treatment because of reporting symptoms on the system? Were you happy with these changes?
- Did anyone else (such as a relative) help you use the system? Do you think they found it useful?

Admissions and calls to the hospital

- Did you need to contact the hospital at any point due to symptoms or side-effects? If so, who did you contact? Did you use eRAPID prior to contacting the hospital? If not, did you

consider using the eRAPID system before you contacted the hospital? Did you use the card in your booklet? How/why did you decide to contact the hospital? How long were you unwell for before you contacted the hospital?

If patient was admitted during their time on study:

- Can you tell us a bit about your admission to hospital and what happened in the lead up to that?
- Did you use the eRAPID system before you contacted the hospital?
- Did the staff on the acute ward mention eRAPID to you, or did you mention it to them?
- Did your admission have any effect on your treatment? (e.g. delays, dose reduction)

If patient had any reported any clinically severe symptoms (triggering advice to contact the hospital)

- When you received the advice to contact the hospital, did you do so? If not, what action did you take and why?
- Did anybody contact you? Did they discuss your eRAPID results with you?
- What were the consequences of that contact?

Supplementary file B: Summary of professional interview schedule

Awareness

- How did you hear about eRAPID/QTool?
- Did you use the symptom report without being prompted by the patient or researcher? If yes, what influenced you to do so?
- What percentage of patients who had eRAPID/QTool results on PPM did you use/view?

Accessing symptom reports in the EPR

- Were you offered any training prior to using the system? Was there anything about the training that could have been done differently? Are you aware that online training is now available?
- Do you have any suggestions how we may improve communication with staff who are using the system?
- How useful did you find the one page prompt guides? (Positive and negative feedback)
- Did you use the facility at the bottom of the results to change access to the number of results you could view? Is there any value to this facility?
- What do you think of the way in which symptoms/adverse events are recorded/ displayed in patient records through the eRAPID system?
- Could you give examples of any positive and negatives experiences you had in accessing the eRAPID results (ease of use) on the EPR?

Consultations

- What do you think the patients think about using eRAPID? Both in terms of logging in/answering the symptom reports and the value of the advice given.
- How has using the system impacted your consultation/assessment with patients?
- Did it change the doctor/nurse/patient relationship in any way? Could you give an example?

How did using the system impact on the length of time of the consultation?

- Were there any times when patient reported symptoms in the consultation did not match reported symptoms on the system? Could you give an example?
- Can you recall occasion/s when using the system influenced a change in patient management or treatment?
- What do you consider were the expected benefits/burdens in using the eRAPID system during the consultation?
- What do you consider were the unexpected benefits/burdens in using the eRAPID system during the consultation?
- What are your thoughts regarding the way in which patients have used the self-management advice available on the eRAPID system?

Severe symptoms notifications

- Have you ever responded to an alert on the system? If so, can you talk me through any particular issues?

General

- Overall, what do you think were the main advantages and disadvantages to using the system?
- Do you have any suggestions for how we could promote/encourage staff to access/use the eRAPID patient data in PPM in the future?
- What do you think the main facilitators were in using the eRAPID system?
- What do you think the main barriers were in using the eRAPID system?
- Do you have any suggestions in how we could improve the system?
- Would you recommend systems like eRAPID to other centres? If yes/no, why?

Supplementary file C: Summary of patient end of study feedback form: Multiple choice items and accompanying response options

1. How easy or difficult was it to learn how to use the eRAPID system?<br>Very easy/Easy/Neither easy nor difficult/Difficult/Very difficult
2. How easy or difficult did you find accessing the system? (e.g. finding the website and logging in)<br>Very easy/Easy/Neither easy nor difficult/Difficult/Very difficult
3. How easy or difficult was it to answer the questions about your symptoms?<br>Very easy/Easy/Neither easy nor difficult/Difficult/Very difficult
4. How did you feel about the amount of time it took to complete the symptom questions?<br>Too long/About right/ Too quick
5. How relevant were the symptom questions to you?<br>Not relevant at all/ Very few questions were relevant/Neither relevant or irrelevant/ Quite relevant/ Very relevant
6. What did you think about completing these questionnaires every week?<br>Definitely too often/ A little bit too often/ Unsure/ I was happy to complete them every week/ I would have been happy to complete them more often
7. Were there any times when you missed a week of completing the symptom questionnaire?<br>No/Yes
8. Did the doctors and nurses you saw during your treatment use your eRAPID symptoms information during consultations?<br>Yes, quite a bit/ Sometimes/ Not at all
9. If yes, did you feel this improved your consultations with the staff?<br>Yes, quite a bit/ Sometimes/ Not at all
10. To what extent do you feel that the symptom questionnaire was useful for the doctors and nurses you saw during your treatment? Very useful/A little useful/Unsure/Not very useful/Not at all useful
11. How useful did you find the information on the eRAPID website about the symptoms and side effects of cancer treatment? Very useful/A little useful/Unsure/Not very useful/Not at all useful
12. Would you recommend the eRAPID system to other cancer patients?<br>No/Not sure/Yes

Supplementary file D: Clinician eRAPID feedback form

Date of completion_________________ Name of clinician_______________________

1. **How well did you know this patient from before?**

Never met him/her before	
A little	
Moderately well	
Very well	

2. **Did you look at the patients' eRAPID symptom information in PPM before/ during the consultation?**

Yes	No

3. **Did you use the eRAPID symptom information in the clinic discussion?**

Very much	Quite a bit	Somewhat	A little	Not at all

4. **Did you find the eRAPID symptom information useful?**

Very much	Quite a bit	Somewhat	A little	Not at all

5. **If yes, in what way?**

**Provided additional information**	
**Confirmed your knowledge of patients' problems**	
**Identified issues/problems to be discussed**	
***Contributed to management**	

****If you answered "Contributed to management", please specify in what way below.***

Change of medication	
Ordering of investigations	
Decision about chemotherapy	
Referral to supportive services (e.g. psycho-oncology, social worker)	
Counselling about lifestyle	

Other: Please specify ☐

6. **Are there any additional ways you have found the eRAPID symptom information useful?**

Supplementary file E: Graphical summary of additional information from clinician feedback forms

FIGURE 1 CLINICIAN FEEDBACK ON WAYS ERAPID WAS USEFUL

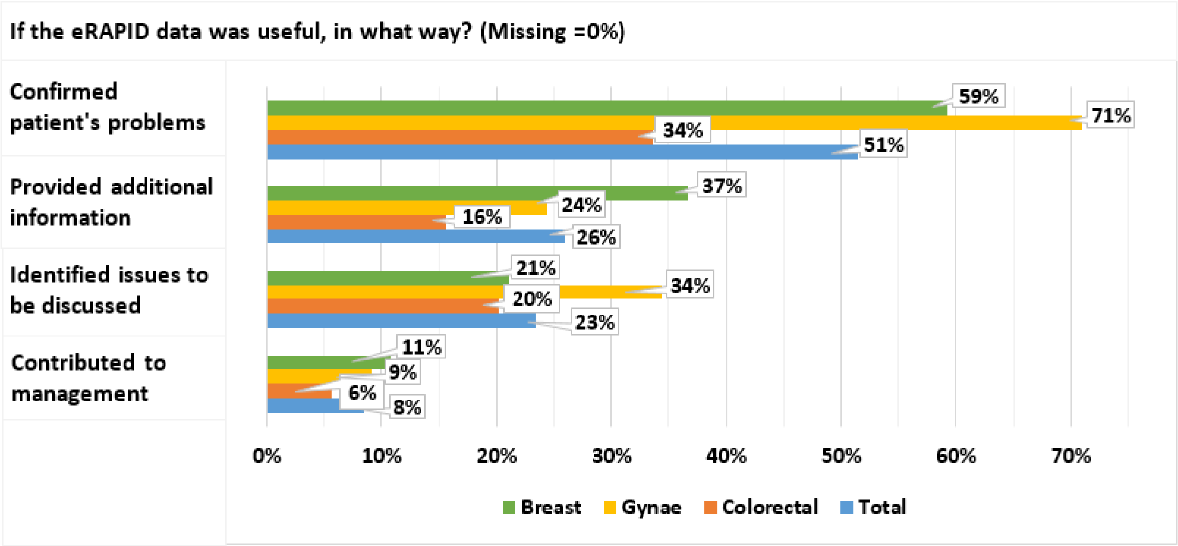

FIGURE 2 CLINICIAN FEEDBACK ON HOW ERAPID CONTRIBUTED TO MANAGEMENT

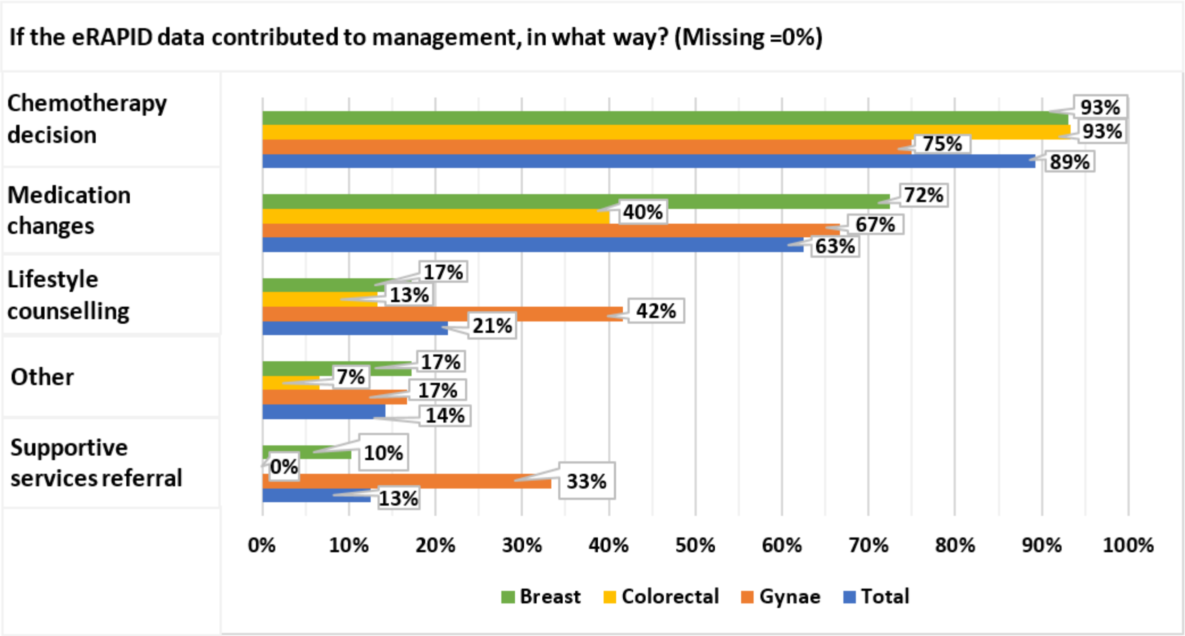

Supplement: online supplemental file 2 [file bmjopen-14-11-s001.pdf]
