# Supplementary material for: Patient and clinician perspectives of an eHealth intervention for supporting cancer treatment in the UK: mixed methods evaluation of the eRAPID randomised controlled trial
Source: BMJ Open. 2024 Nov 7;14(11):e078283. doi: 10.1136/bmjopen-2023-078283 (PMC11552561; doi:10.1136/bmjopen-2023-078283)
Supplement: Supplementary file 2 [file bmjopen-2023-078283.reviewer_comments.pdf]

PEER REVIEW HISTORY

BMJ Open publishes all reviews undertaken for accepted manuscripts. Reviewers are asked to complete a checklist review form and are provided with free text boxes to elaborate on their assessment. These free text comments are reproduced below.

ARTICLE DETAILS

Title (Provisional)

Patient and clinician perspectives of an eHealth intervention for supporting cancer treatment in the UK: Mixed methods evaluation of the eRAPID randomised controlled trial

Authors

Warrington, Lorraine; Holmes, Marie; Gibson, Andrea; Peacock, Rosemary; Rogers, Zoe; Dickinson, Sarah; Holch, Patricia; Hewison, Jenny; Hulme, Claire; Dawkins, Bryony; Woroncow, Barbara; Cucchi, Virginia; Hudson, Eleanor Mae; Brown, Julia; Velikova, Galina; Absolom, Kate

---

VERSION 1 - REVIEW

**Reviewer**	**1**
**Name**	**Chambers, Pinkie**
**Affiliation**	**University College London, Practice and Policy**
**Date**	**27-Oct-2023**
**COI**	**none**

Thank you for the opportunity to review this review this manuscript. Overall the paper is really nicely written . I believe the work will be helpful with other evidence produced to support implementation.

I have minor revisions that would strengthen the work.

1. Abstract: 1st line states "an RCT" - please check all abbreviations are written in full the first time they are mentioned. Double check it is an RCT or a RCT?

2.Methods subtitle : Summary of feedback obtained from patients and clinicians - do you mean Procedures of feedback. Summary of feedback implies a result rather than a method.

3. Questionnaire and interview question development. There is no mention of how these questions were developed, tested or validated. Please could this be added.

4. Results - It was great that so many completed the questionnaire 186/222 (84%) . Were these 100% of patients that were on treatment at the end of the period. I wasn't clear. It was implied but not that explicit.

5. Conclusions - This should be labelled discussion

6.Really important points were made in the discussion. I wonder if the gynae clinicians were more engaged because of the toxicities of some treatments being less measured but more reported i.e. peripheral neuropathy or nausea and vomiting. Lastly, would implementation be improved through stratification and

---

**Reviewer**	**2**
**Name**	**Wolff, Angela C.**
**Affiliation**	**Trinity Western University**
**Date**	**08-Nov-2023**
**COI**	**none**

ALSO ATTACHED

Abstract

Please edit abstract based on the suggested changes made below regarding the body of the paper.

Paragraph prior to method & Method section

p. 7, line 52-54 would be more appropriately stated as the study design is a mixed method approach, specifically an explanatory sequential design (three-phase design) (Leavy, 2023). This would need to be aligned further with the content on p. 8. The first phase was the RCT, followed by and the second phase was both qualitative descriptive [Bradshaw et al., 2017] and survey design. As per reporting standards for mixed approaches, a diagram should be included to illustrate the phases.

• Leavy, P. (2023). Research design quantitative, qualitative, mixed methods, arts-based, and community-based participatory research approaches (2nd ed.). Guildford Publications.

• Bradshaw, C., Atkinson, S., & Doody, O. (2017). Employing a qualitative description approach in health care research. Global Qualitative Nursing Research, 4, 1-8. Doi: 10.1177/2333393617742282

No research questions provided, assume not required by journal.

p. 7 line 54-56 consider rewording to align with the purpose of qualitative interviews and surveys as being descriptive about the experiences of patients and clinicians. For example, “interviews and surveys were used describe the experiences and elicit views from patients and clinicians about the intervention and …” This would align with the nature of the research question you were seeking to answer and the aim statement. Line 54, is it to explore or describe, or both? As well, with the aim statement if the primary outcome of eRapid was symptom control and secondary was the impact of the measures how does that align with

the questions asked in the interviews and survey? Revise for congruency with the outcomes and the interview and survey questions detailed on p. 8 lines 29-31, 35-36 and p. 15 line 3-4.

p. 7 line 52, define what is meant by “acceptability” and “clinical value”, it seems to be operationalized to included usability, benefits of certain features, value/relevance.

On p. 8 line 1 it would be helpful to state the mixed approach and design to then identify the RCT portion and then be explicit as to the research designs for the 2nd phase. Elaborate as to the importance of using a mixed approach and then rationale for two designs to elicit feedback.

Line 25, p. 8 it is unclear if the interviews are at 18-weeks.

Line 43 p. 8 The purposive sampling used for the interviews is clearly articulated, however, the type of sampling for the feedback questionnaire should be included. Also, how was recruitment conducted for both.

The updates should also be reflected in the abstract

Page 7-8 the use of survey and questionnaire are used, is it survey design and then a questionnaire was sent out?

p. 8 line 40 elaborate further that Feedback was elicited from patients and clinicians using two different questionnaire that were developed by the researchers (?). Indicate both collected at 18-weeks and discuss sampling and recruitment. Also, please indicate the range of time it took to complete, similar to reporting the length of the interviews, which should be reported as well on p. 8 line 26.

p. 9 line 26 for the method of analysis I would suggest stating thematic analysis is the approach used. Also, there is a newer publication, however, unsure if you analyses match the newer publication. Perhaps review. Braun, V., & Clarke, V. (2022). Conceptual and design thinking for thematic analysis. Qualitative Psychology, 9(1), 3-26. https://doi.org/10.1037/qup0000196

p. 9 Line 29 and 34 I believe you are referring to the themes generated as a “framework”. Please clarify.

p. 9 line 34 and 39-40 please clarify the difference between the 2 statements. For the “joint display approach” reference, please provide a citation in reference to the approach used.

p. 9 line 43 can you provide a reference for “joint display approach” or explain what is meant by this. Perhaps refer back to the sequential mixed methods design and integration of findings from RCT quant, survey, and qual interview data.

p. 9 line 44-45 you indicate the primary and secondary outcome reported, which are also in Table 2. Is there any benefit in referring to the table at this point in the paper? This way the reader does not feel compelled to retrieve the paper.

Results

p. 10 lines 16-19 ensure they are consistent with the headings used for this section on lines 25 and p. 11. Same with the heading for the clinician perspectives p. 12-13

for the statistical analyses please report confidence intervals (p. 10 line 33-34). P. 13 lines 21-30, was statistical testing done to examine this difference?

p. 10 line 40 could you provide a few examples in brackets after the statement about improvements in addition to the app.

p. 10-14, I appreciate the qualitative results and to further emphasize the meaning of each theme, it might be advantageous to start off with a topic sentence for each paragraph to provide the gist of the theme to your readers (first sentence after each heading and subheading). This can quickly orientate the reader to the finding and then you present the finding. … lines 27… should there be a general sentence to introduce what this theme refers to. To create flow in the reporting of the patients' and clinicians' perspectives you may want to consider placing all the quotes in a table. This would then allow for the use of topic and transition sentences in each paragraph as you talk about the qualitative findings.

p. 11 line 27 did the patients "think" or "felt" – consider alternate wording for felt and line 46 was it an "experience" or a "feeling"? Also p. 15 line 8 & 9.

p. 14 line 1 clinicians reporting should be clinicians reported, line 16 included rather than including (past tense used to report findings of a study).

p. 14 line 24-25 should read '… map these with experiences described by patient and clinicians from end-of-study interviews and questionnaires."

p. 14, line 30 to 31 clarify the meaning of "limitations of benefits" and the example should be placed into brackets or written as a sentence

p. 14 line 50 does "clinician feedback forms" refer to the questionnaire.

On p. 14 the meta-integration is an important part of a mixed methods study. I would suggest placing the comments from the findings in a table, and then expanding this section further to integrate not only the interview data but also the questionnaire data. It is not evident how the clinician feedback has been integrated with the RCT portion.

Table 2, for the first column on the key findings from the RCT can you provide the statistical findings in brackets behind the finding? Also, for the RCT findings you probably require a citation – perhaps the first column heading (key findings from RCT – add citation).

Discussion

The discussion is clear and focused to relate back to the purpose of the mixed method study. My only comment for consideration is also the need for more computer adaptive testing to ensure that patients receive the relevant questions and the right time to meet their needs. This could reflect the need for person-centered outcome assessments where at different stages of the cancer trajectory certain questions are more relevant than others. This would

acknowledge that the same PROMs are not appropriate for all people and/or questions are adapted for different people due to health inequities.

Figures

Given that the patient responses were in the body of the paper, consider also placing the clinicians responses in the body of the paper rather than supplementary data. Unsure if this was a page limit issue but it would make sense to have both in the paper.

VERSION 1 - AUTHOR RESPONSE

**Reviewer 1 (PC)**	**Comment from authors**
1. Abstract: 1st line states "an RCT" - please check all abbreviations are written in full the first time they are mentioned. Double check it is an RCT or a RCT?	We have now added the abbreviated term in full and checked through the full document to ensure we have done this consistently elsewhere. For terminology - both 'an RCT' and 'a RCT' seem to be acceptable, but 'an RCT' is used more frequently (though of course it is 'a randomised controlled trial'.<br><br>*(Throughout all sections)*
2. Methods subtitle: Summary of feedback obtained from patients and clinicians - do you mean Procedures of feedback. Summary of feedback implies a result rather than a method.	We have now changed this heading to 'Procedures for obtaining feedback from patients and clinicians', which we hope is clearer.<br><br>*(Section: Methods>Procedures for obtaining feedback from patients and clinicians*
3. Questionnaire and interview question development. There is no mention of how these questions were developed, tested or validated. Please could this be added.	We have added some additional detail on the development of the questionnaires and interview schedules, including references to where these have been used in previous work.<br><br>*(Section: Methods>Procedures for obtaining feedback from patients and clinicians, paragraphs 1, 3 and 4)*
4. Results - It was great that so many completed the questionnaire 186/222 (84%). Were these 100% of patients that were on treatment at the end of the period. I wasn't clear. It was implied but not that explicit.	There were 222 patients still on study at 18 weeks and 186 completed the feedback questionnaires. We have made some changes to wording in the method and results sections to clarify this.<br><br>*(Section: Results>Participants>Patient sample, paragraph 1)*
5. Conclusions - This should be labelled discussion	Thank you- we have now changed this.<br><br>*(Section: Discussion)*

6.Really important points were made in the discussion. I wonder if the gynae clinicians were more engaged because of the toxicities of some treatments being less measured but more reported i.e. peripheral neuropathy or nausea and vomiting. Lastly, would implementation be improved through stratification and	We apologise but as some of the text of this comment was missing, we were unsure of how to address the second part about implementation.<br><br>In terms of the gynaecological clinical team having higher levels of engagement with eRAPID it was not apparent from the feedback that this was driven by expected worse toxicity and symptomatology in this patient group- (though many of the ovarian cancer patients live with advanced disease and complex health needs). Anecdotally through the research team's experience in designing and delivering the trial the gynaecology specialist nursing team were very supportive throughout.<br><br>We are planning further work outside the scope of this paper to explore the symptom reported data collected through this trial to understand symptom profiles and trajectories across the cancer groups.
**Reviewer 2 (AW)**	**Comments from authors**
Abstract - Please edit abstract based on the suggested changes made below regarding the body of the paper.	Thank you, we have updated the abstract to reflect changes made.<br><br>*(Section: Abstract)*
Paragraph prior to method & Method section p. 7, line 52-54 would be more appropriately stated as the study design is a mixed method approach, specifically an explanatory sequential design (three-phase design) (Leavy, 2023). This would need to be aligned further with the content on p. 8. The first phase was the RCT, followed by and the second phase was both qualitative descriptive [Bradshaw et al., 2017] and survey design. As per reporting standards for mixed approaches, a diagram should be included to illustrate the phases.<br>• Leavy, P. (2023). Research design quantitative, qualitative, mixed methods, arts-based, and community-based participatory research approaches (2nd ed.). Guildford Publications.<br>• Bradshaw, C., Atkinson, S., & Doody, O. (2017). Employing a qualitative description approach in health care research. Global Qualitative Nursing Research, 4, 1-8. Doi: 10.1177/2333393617742282	We thank the reviewer for this comment. We have now included additional detail on the methodology used. In summary, the collection of the qualitative and wider survey feedback data were embedded into the RCT methodology. The analysis of the main RCT data and the qualitative/survey feedback were conducted simultaneously following the completion of the trial. Therefore, our approach was a concurrent triangulation design and we have explained this by adding a new figure outlining the design and process (Figure 1) and adding a short paragraph to the beginning of the methods section with a reference to this approach (Ref 22: Creswell, J.W. and Plano Clark, V.L. (2011) Designing and Conducting Mixed Methods Research. 2nd Edition, Sage Publications, Los Angeles)<br><br>*(Section: Methods, paragraph 1)*
No research questions provided, assume not required by journal.	This is not a specified requirement of the journal but (as described below) we have amended the introduction to be clearer on the specific aims of this mixed methods approach.

	(Section: Aims and objectives)
p. 7 line 54-56 consider rewording to align with the purpose of qualitative interviews and surveys as being descriptive about the experiences of patients and clinicians. For example, "interviews and surveys were used describe the experiences and elicit views from patients and clinicians about the intervention and …" This would align with the nature of the research question you were seeking to answer and the aim statement. Line 54, is it to explore or describe, or both? As well, with the aim statement if the primary outcome of eRapid was symptom control and secondary was the impact of the measures how does that align with the questions asked in the interviews and survey? Revise for congruency with the outcomes and the interview and survey questions detailed on p. 8 lines 29-31, 35-36 and p. 15 line 3-4.	We have made some changes to the introduction and separated out the aims to make it clearer to the reader what we wanted to achieve with the mixed methods approach.<br><br>*(Section: Aims and objectives)*
p. 7 line 52, define what is meant by "acceptability" and "clinical value", it seems to be operationalized to included usability, benefits of certain features, value/relevance.	We have now rewritten this section to make the aims clearer.<br><br>*(Section: Aims and objectives)*
On p. 8 line 1 it would be helpful to state the mixed approach and design to then identify the RCT portion and then be explicit as to the research designs for the 2nd phase. Elaborate as to the importance of using a mixed approach and then rationale for two designs to elicit feedback.	We have now added some additional detail on this through the introduction and method section which we believe makes this clearer.<br><br>*(Section: Aims and objectives, see also Methods, paragraph 1)*
Line 25, p. 8 it is unclear if the interviews are at 18-weeks.	The interviews were conducted around the 18 week/end of study time point and this has now been clarified in the text.<br><br>*(Section: Methods>Procedures for obtaining feedback from patients and clinicians, Interview procedures, paragraph 1)*
Line 43 p. 8 The purposive sampling used for the interviews is clearly articulated, however, the type of sampling for the feedback questionnaire should be included. Also, how was recruitment conducted for both.	We have now added some additional detail on this.<br><br>*(Section: Methods>Procedures for obtaining feedback from patients and clinicians, Feedback questionnaire procedures)*
The updates should also be reflected in the abstract	We have updated the abstract to reflect changes and comments.<br><br>*(Section: Abstract)*
Page 7-8 the use of survey and questionnaire are used, is it survey design and then a questionnaire was sent out?	We have made some changes to be more consistent in our terminology describing the feedback questionnaires.

	(Throughout all sections)
p. 8 line 40 elaborate further that Feedback was elicited from patients and clinicians using two different questionnaire that were developed by the researchers (?). Indicate both collected at 18-weeks and discuss sampling and recruitment. Also, please indicate the range of time it took to complete, similar to reporting the length of the interviews, which should be reported as well on p. 8 line 26.	The clinician questionnaires were collected throughout the study period and not just at 18 weeks. We have added a figure and additional text to make this clearer.<br>The researchers were not generally present when patients and clinicians completed the feedback forms and the time completed was not recorded.<br><br>*(Section: Methods>Procedures for obtaining feedback from patients and clinicians, Feedback questionnaire procedures>Clinician feedback questionnaire, paragraph 1)*
p. 9 line 26 for the method of analysis I would suggest stating thematic analysis is the approach used. Also, there is a newer publication, however, unsure if you analyses match the newer publication. Perhaps review. Braun, V., & Clarke, V. (2022). Conceptual and design thinking for thematic analysis. Qualitative Psychology, 9(1), 3-26. https://doi.org/10.1037/qup0000196	We used a framework method for analysis, which is a specific type of qualitative analysis which comes under the bracket of thematic analysis. We have added a short explanation and justification for this, in addition to another reference. We have also now updated the Braun & Clarke reference.<br><br>*(Section Analysis>Qualitative data (interviews and free text written comments, paragraph 1)*
p. 9 Line 29 and 34 I believe you are referring to the themes generated as a “framework”. Please clarify.	We have added some additional detail and a reference for the framework method used.<br><br>*(Section Analysis>Qualitative data (interviews and free text written comments, paragraph 1)*
p. 9 line 34 and 39-40 please clarify the difference between the 2 statements. For the “joint display approach” reference, please provide a citation in reference to the approach used.	We have made some changes to clarify this and added another reference for the joint display approach.<br><br>*(Section: Analysis, all paragraphs, refs 21, 29)*
p. 9 line 43 can you provide a reference for “joint display approach” or explain what is meant by this. Perhaps refer back to the sequential mixed methods design and integration of findings from RCT quant, survey, and qual interview data.	We have added another reference for the joint display approach. We have also added a figure to help illustrate the sources of data and how they were integrated.<br><br>*(Section: Analysis>Synthesis of participant feedback with main RCT findings, Refs 21, 29, Figure 1)*
p. 9 line 44-45 you indicate the primary and secondary outcome reported, which are also in Table 2. Is there any benefit in referring to the table at this point in the paper? This way the reader does not feel compelled to retrieve the paper.	We have added this.<br><br>*(Section: Analysis>Synthesis of participant feedback with main RCT findings)*
Results	

p. 10 lines 16-19 ensure they are consistent with the headings used for this section on lines 25 and p. 11. Same with the heading for the clinician perspectives p. 12-13	Thank you for bringing our attention to these inconsistencies. These have now been changed.<br><br>*(Throughout all sections)*
for the statistical analyses please report confidence intervals (p. 10 line 33-34). P. 13 lines 21-30, was statistical testing done to examine this difference?	Confidence intervals have now been added. For the differences between clinical groups (P13, lines 21-30), this data is descriptive only and due to small cell sizes in some frequencies, we do not feel that statistical testing is appropriate.<br><br>*(Section: Results>Patient perspectives>Acceptability and functionality>Ease of Use, paragraph 1)*
p. 10 line 40 could you provide a few examples in brackets after the statement about improvements in addition to the app.	We have now added some additional examples.<br><br>*(Section: Results>Patient perspectives>Acceptability and functionality>Ease of Use, paragraph 3)*
p. 10-14, I appreciate the qualitative results and to further emphasize the meaning of each theme, it might be advantageous to start off with a topic sentence for each paragraph to provide the gist of the theme to your readers (first sentence after each heading and subheading). This can quickly orientate the reader to the finding and then you present the finding. … lines 27… should there be a general sentence to introduce what this theme refers to. To create flow in the reporting of the patients' and clinicians' perspectives you may want to consider placing all the quotes in a table. This would then allow for the use of topic and transition sentences in each paragraph as you talk about the qualitative findings.	We thank the reviewer for this suggestion, which we have partially implemented. We have now included short descriptions of each theme. We have not included this for subthemes as we felt it reduced flow and readability.<br><br>*(Throughout section Results)*
p. 11 line 27 did the patients "think" or "felt" – consider alternate wording for felt and line 46 was it an "experience" or a "feeling"? Also p. 15 line 8 & 9.	We have made some changes throughout the document to reword using 'felt' as a descriptor, other than those where patients explicitly reported 'feeling' something - e.g. feeling empowered.<br><br>*(Throughout all sections)*
p. 14 line 1 clinicians reporting should be clinicians reported, line 16 included rather than including (past tense used to report findings of a study).	We have now changed this.<br><br>*(Section: Results>Clinician perspectives, Impact on clinical care, paragraph 7)*
p. 14 line 24-25 should read '… map these with experiences described by patient and clinicians from end-of-study interviews and questionnaires."	We have revised this- the clinician questionnaires were not at the end of the study so the new wording now reflects this.<br><br>*(Section: Results>Synthesis of feedback with key findings from the eRAPID RCT, paragraph 1)*
p. 14, line 30 to 31 clarify the meaning of "limitations of benefits" and the example	We have now changed the wording to make it clearer that this refers to the benefits being limited to the earlier timepoints (6 and 12 weeks).

should be placed into brackets or written as a sentence	*(Section: Results>Synthesis of feedback with key findings from the eRAPID RCT, paragraph 2)*
p. 14 line 50 does "clinician feedback forms" refer to the questionnaire.	Yes, this does refer to the questionnaire. We have worked through the document to ensure we are consistent with the wording we use.<br><br>*(Throughout all sections)*
On p. 14 the meta-integration is an important part of a mixed methods study. I would suggest placing the comments from the findings in a table, and then expanding this section further to integrate not only the interview data but also the questionnaire data. It is not evident how the clinician feedback has been integrated with the RCT portion.	In Table 2, we have summarised the themes and how these apply to the RCT. We had previously tried presenting this with relevant quotes and comments but found that it was much easier to understand and read when presented with the general themes. The primary and secondary outcomes of the RCT focused on evaluating benefits for patients and as such, there is more integration with patient data. However, although the clinician views and experiences do not map as directly onto the RCT results, they certainly will have impacted on these, particularly the important finding that patients adherence was associated with clinician use of eRAPID and we have emphasised this where possible.<br>Similarly, the data from feedback questionnaires was important to describe the overall views of eRAPID, but the interview data provided more nuanced insights. We have added some content to the discussion briefly outlining this.<br><br>*(Section: Results, Table 2, see also Discussion, paragraph 8)*
Table 2, for the first column on the key findings from the RCT can you provide the statistical findings in brackets behind the finding? Also, for the RCT findings you probably require a citation – perhaps the first column heading (key findings from RCT – add citation).	Thank you, have now added the citation for the RCT in the heading to help navigate the reader to the main trial results paper. On balance we felt it would be confusing to add all statistical findings to this, due the variety of methods used in the statistical analysis.<br><br>*(Table 2)*
Discussion	
The discussion is clear and focused to relate back to the purpose of the mixed method study. My only comment for consideration is also the need for more computer adaptive testing to ensure that patients receive the relevant questions and the right time to meet their needs. This could reflect the need for person-centered outcome assessments where at different stages of the cancer trajectory certain questions are more relevant than others. This would acknowledge that the same PROMs are not appropriate for all people and/or questions	The relevance of PROMS for both clinicians and patients is crucial and was an important part of the development work for eRAPID. We have now added some content to the discussion to reflect this.<br><br>*(Section: Discussion, paragraph 10)*

are adapted for different people due to health inequities.	
Figures	
Given that the patient responses were in the body of the paper, consider also placing the clinicians responses in the body of the paper rather than supplementary data. Unsure if this was a page limit issue but it would make sense to have both in the paper.	The main clinician responses are in the body of the paper (Figure 3). The clinician responses in the supplementary file were responses to sub-questions and were put here due to limitations on the number of figures permitted in the main paper. We have made this more explicit in the text.<br><br>*(Section: Results>Clinician perspectives, Impact on clinical care, paragraph 4)*

VERSION 2 - REVIEW

Reviewer **1**

Name **Chambers, Pinkie**

Affiliation **University College London, Practice and Policy**

Date **10-Sep-2024**

COI **None**

I am happy with the corrections the author has made.

Reviewer **2**

Name **Wolff, Angela C.**

Affiliation **Trinity Western University**

Date **21-Sep-2024**

COI **I have co-presented two workshops with Kate Absolom. I know both Kate and Galina Velikova through the International Society of Quality of Life.**

Abstract

The design should be specified in the “design and participants” section. Does the journal require this section to be titled Methods?

Other

There are several spelling and grammatical errors (including duplication of words) throughout the manuscript.

• p. 7 line 21 “growing”

• p. 8 line 4 “exploreE”

• p. 9 line 8 “this”

• p. 10 line 48 “acceptabilityA”

• and many more from p. 11 onwards

• extra spaces between words with 2 spaces rather than 1 space (e.g., p. 9 line 9).

Throughout the document, there is incorrect punctuation, with periods duplicated before and after citations (e.g, p. 7 line 59, p. 9 line 31, p. 10 line 19) and participant quotes (e.g., line 13 line 23). Fix according to journal specifications.

Introduction

p. 7, first paragraph you refer to the use of PROMs to monitor issues. Keep consistent with this term. Line 15 states “recall of problems experienced”. Change from problems to issues.

p. 7, lines 38-43 please indicate which of these are PROMs.

p. 7 line 52 please provide the refer after the sentence ending with “symptom control.”

p. 8 line 4 rather than “professional” use “clinician” so the terminology aligns with how the data is reported.

Methods

p. 8 line 26 rather than a “+” use “and”

p. 9 line 3 finish the sentence with “feedback through questionnaires”

Results

Several sentences start with numerical percentages, the numbers should be spelled out in full. E.g., p. 10 line 29; p. 11 line 52; p. 12 line 11.

p. 11, line 41 add the word clinicians to “and clinicians not having symptoms data…”

p. 13 line 39 combine with the previous sentence on line 37 to form one paragraph. Same with p. 15 line 3-5 to form one paragraph.

p. 15 lines 46-57 do not make sense given the headings. Please consider rewriting to convey your findings.

Discussion

p. 16 lines 47-51, first sentence of the paragraph is a run-on sentence.

p. 17 line 24 first time used the abbreviation IT so spell in full.

p. 17 line 42 instead of “clinical teams” with “clinicians”

VERSION 2 - AUTHOR RESPONSE

We thank the Editor and reviewers for their helpful feedback. We summarise how these have been addressed in the attached word file.

BMJ Open Manuscript ID bmjopen-2023-078283.R1
Response to Reviewer comments- 3rd October 2024

**Reviewer: 1**<br>Dr. Pinkie Chambers, University College London	
Comments to the Author:<br>I am happy with the corrections the author has made.	Many thanks for your comments.
**Reviewer: 2**<br>Dr. Angela C. Wolff, Trinity Western University<br>Comments to the Author:	
Abstract	
The design should be specified in the “design and participants” section. Does the journal require this section to be titled Methods?	We have retitled this section methods and added the design.
Other	
There are several spelling and grammatical errors (including duplication of words) throughout the manuscript.<br>• p. 7 line 21 “growing”<br>• p. 8 line 4 “exploreE”<br>• p. 9 line 8 “this”<br>• p. 10 line 48 “acceptabilityA”<br>• and many more from p. 11 onwards<br>• extra spaces between words with 2 spaces rather than 1 space (e.g., p. 9 line 9).<br>Throughout the document, there is incorrect punctuation, with periods duplicated before and after citations (e.g, p. 7 line 59, p. 9 line 31, p. 10 line 19) and participant quotes (e.g., line 13 line 23). Fix according to journal specifications.	We apologise these errors were left in the marked-up copy of the manuscript.<br>They were however, not present in the clean version of the manuscript.<br>We have made all changes in this current round to the clean version of the manuscript, so these errors should no longer be present.
Introduction	
p. 7, first paragraph you refer to the use of PROMs to monitor issues. Keep consistent with this term. Line 15 states “recall of problems experienced”. Change from problems to issues.	We have changed this to be more consistent throughout the manuscript.
p. 7, lines 38-43 please indicate which of these are PROMs.	We have added this detail to clarify which are PROMs and which data was collected from hospital records.
p. 7 line 52 please provide the refer after the sentence ending with “symptom control.”	This has now been moved/added.

p. 8 line 4 rather than "professional" use "clinician" so the terminology aligns with how the data is reported.	This has been changed throughout the manuscript to be more consistent.
Methods	
p. 8 line 26 rather than a "+" use "and"	This has now been changed, although we have used the word 'plus' rather than 'and' for clarity.
p. 9 line 3 finish the sentence with "feedback through questionnaires"	This has now been changed.
Results	
Several sentences start with numerical percentages, the numbers should be spelled out in full. E.g., p. 10 line 29; p. 11 line 52; p. 12 line 11.	This has now been changed throughout.
p. 11, line 41 add the word clinicians to "and clinicians not having symptoms data…"	This has been added.
p. 13 line 39 combine with the previous sentence on line 37 to form one paragraph. Same with p. 15 line 3-5 to form one paragraph.	This has been changed.
p. 15 lines 46-57 do not make sense given the headings. Please consider rewriting to convey your findings.	We have now rephrased this for clarity.
Discussion	
p. 16 lines 47-51, first sentence of the paragraph is a run-on sentence.	We have rephrased this.
p. 17 line 24 first time used the abbreviation IT so spell in full.	This has been added.
p. 17 line 42 instead of "clinical teams" with "clinicians"	This has been changed.
